# Prognostic significance of mast cell and microvascular densities in malignant peripheral nerve sheath tumor with and without neurofibromatosis type 1

**DOI:** 10.1002/cam4.1977

**Published:** 2019-02-08

**Authors:** Roberto André Torres de Vasconcelos, Pedro Guimarães Coscarelli, Thyago Marsicano Vieira, Washington Silva Noguera, Davy Carlos Mendes Rapozo, Marcus André Acioly

**Affiliations:** ^1^ Division of Bone and Connective Tissue, Department of Surgical Oncology National Cancer Institute Rio de Janeiro Brazil; ^2^ Postgraduation Program in Neurology Federal University of the State of Rio de Janeiro Rio de Janeiro Brazil; ^3^ Division of Internal Medicine State University of Rio de Janeiro Rio de Janeiro Brazil; ^4^ Division of Pathology National Cancer Institute Rio de Janeiro Brazil; ^5^ Division of Neurosurgery Federal University of Rio de Janeiro Rio de Janeiro Brazil; ^6^ Division of Neurosurgery Fluminense Federal University Niterói, Rio de Janeiro Brazil

**Keywords:** malignant peripheral nerve sheath tumor, mast cell, mast cell density, microvascular density, neoangiogenesis, neurofibromatosis type 1

## Abstract

Malignant peripheral nerve sheath tumors (MPNSTs) are rare and aggressive soft tissue sarcomas with a significant susceptibility to metastasize early in their course. Pathogenesis is yet to be fully elucidated. Recently, the essential role of mast cells in the tumor onset of neurofibromatosis type 1 (NF1)‐associated neurofibromas and MPNSTs was confirmed in both experimental and human studies. In this study, we investigate mast cell density (MCD), microvascular density (MVD), and proliferation index (Ki‐67) in MPNST. A secondary aim was to correlate histological staining to clinical data and survival in patients with and without NF1. In total, 34 formalin‐fixed paraffin‐embedded MPNST tissues from 29 patients were eligible. MCD, MVD, and Ki‐67 labeling index (LI) were analyzed in all stained tissues by a computer‐based quantitative algorithm (Aperio ImageScope). In addition, chart review was performed for clinical data and survival analysis. Overall, MCD, MVD, and Ki‐67 LI were evenly distributed throughout tumor tissue. There was a negative correlation of NF1 status (affected, *P* = 0.037), tumor size (>10 cm, *P* = 0.023), and MVD in the tumor periphery (higher tercile, *P* = 0.002) to survival. Multivariate analysis confirmed the association of MVD in the tumor periphery (higher tercile, *P* = 0.019) with a decreased overall survival. Diverse mast cell and microvascular distributions suggest that angiogenesis in MPNST occurs independently. The role of mast cells in tumor progression is unclear and lacks prognostic value. Higher MVD has prognostic significance with possible therapeutic implications in MPNST.

## 1 INTRODUCTION

Malignant peripheral nerve sheath tumor (MPNST) is a rare malignant neoplasm, which corresponds to 2%‐10% of all soft tissue sarcomas.^1^, ^2^, ^3^ These tumors mainly affect adults with a median age of 35‐44 years and rarely affect children.^2^, ^4^, ^5^, ^6^ These are aggressive tumors with a significant susceptibility to metastasize early in their course.^4^, ^5^, ^6^

MPNSTs have a widely recognized association with neurofibromatosis type 1 (NF1) in approximately 50% of all cases.^2^, ^4^, ^5^, ^6^, ^7^, ^8^ Such association is of note since MPNST is the main cause of death in such patients.^9^ Surgery is still the mainstay of treatment and the only chance of cure. Extensive tumor resection is the main goal, although not always possible due to the difficult management of tumors in challenging locations, which causes frequent local recurrence and consequently the occurrence of distant metastases.^6^ Generally, the 5‐year overall survival (OS) is about 44%.^8^

MPNSTs have a complex karyotype, showing great genetic variability with expression of multiple oncogenes and loss of tumor suppressor genes.^10^, ^11^, ^12^, ^13^ This usually accounts for the failure of the chemotherapeutic regimens and limits the findings of potentially new molecular therapeutic targets.^14^, ^15^, ^16^ The study of tumor microenvironment in MPNSTs emerges in this context, since it has an important role for several other neoplasms. Some therapeutic approaches directed to non‐neoplastic cells are currently available demonstrating promising results in advanced melanomas by blocking T‐cell regulatory function (ipilimumab and nivolumab),^17^, ^18^ in renal cell carcinomas by blocking angiogenesis,^19^ and in solid tumors by preventing bone events related to bone metastasis (denosumab).^20^

Recently, there has been an increasing interest in the role of mast cells in the tumor microenvironment and its potential use as a target for cancer therapies.^19^, ^21^, ^22^, ^23^, ^24^, ^25^, ^26^, ^27^, ^28^ Mast cells assist in the coordination of a local inflammatory response that ultimately leads to remodeling of the extracellular matrix, to immune response modulation, and also to tumor‐induced neoangiogenesis. They infiltrate tumor microenvironment stimulated by the stem cell factor (SCF), which is secreted by both tissue and neoplastic cells.^29^, ^30^, ^31^, ^32^

Mast cells can promote or protect against tumor onset and progression, which can be defined by the specific interaction with local microenvironment.^26^ For pulmonary adenocarcinomas,^25^ mast cell accumulation coupled with angiogenesis contributes to tumor progression and reduced survival. Conversely, the occurrence of stromal mast cells in breast carcinoma was associated to a better survival.^27^ For renal cell carcinomas, the prognostic significance of mast cell density (MCD) and angiogenesis is still controversial.^19^

In NF1, Yang et al^33^ demonstrated in experimental models that the pathogenesis of cutaneous and plexiform neurofibromas involved not only the loss of heterozygosity of the *NF1* gene (*Nf1 −/−*) in Schwann cells, but was also necessarily associated to a microenvironment composed of haploinsufficient cells (*Nf1 +/−*), especially mast cells, which could be more sensitive to the mediators secreted by tumor cells. Thus, tumoral Schwann cells of the plexiform neurofibroma promote SCF secretion and therefore induce *NF1* haploinsufficient mast cell accumulation and activation. Mast cells gain increased motility and consequently release inflammatory mediators, such as VEGF, heparin, histamine, which are potent pro‐angiogenic factors; and metalloproteinases for remodeling of the extracellular matrix. These changes create a favorable microenvironment to neurofibroma progression and possibly to sarcomatous degeneration into MPNST.^33^

The involvement of mast cells in the pathogenesis and progression of diffuse and encapsulated neurofibromas in patients affected by NF1 was suggested by Tucker et al.^34^ MCD was positively correlated to tumor overall vascularity, even though blood vessels were evenly distributed throughout tumors.^34^ Friedrich et al^35^ have shown that, compared to NF1‐associated and sporadic neurofibromas, MPNST has increased microvascular density (MVD) with decreased mast cell infiltration. The role of mast cell infiltration in MPNSTs was largely unknown until recently, when Dodd et al^36^ demonstrated an accelerated tumor onset in experimental models and human tissues with elevated levels of hematopoietic cells.^36^

The clinical significance of the interaction mast cell/MPNST tumor microenvironment is yet to be determined, however. In this study, we investigate MCD, MVD, and Ki‐67 labeling index (LI) in MPNST. A secondary aim was to correlate histological staining to clinical data and survival in patients with and without NF1. Herein, we show for the first time that MCD is not related to MVD due to different distribution. On the other hand, higher MVD distinguish a subpopulation of MPNST who carry a significant worse prognosis.

## 2 METHODS

### 2.1 Patients and tumors

Between January 1990 and December 2010, 92 consecutive patients were admitted at the National Cancer Institute (INCA, Rio de Janeiro, Brazil) with the diagnosis of MPNST. The epidemiological, clinical, and therapeutic characteristics were previously published.^37^ The histological diagnosis was confirmed by experienced institutional pathologists and reviewed for this study (TMV, WSN). Tumors were considered NF1‐associated if the patient had the clinical diagnosis of NF1, based on two or more of the National Institutes of Health criteria.^38^ The exclusion criteria were as follows: patients who underwent incisional biopsy or neoadjuvant treatment (chemo‐ or radiation therapy [RT] prior to surgery) or patients with unavailable clinical or histopathological data.

A retrospective chart review of patient, tumor, and treatment characteristics was performed. Clinical data included age, gender, NF1 status, tumor location (head and neck, trunk, and extremities), tumor size (maximal diameter), disease stage (American Joint Committee on Cancer staging system),^39^ surgical resection, use of chemotherapy and/or RT, and OS. The institutional review board approved the terms and conditions of the present study (n. 942.009/2011).

### 2.2 Histological evaluation and immunohistochemical staining

Formalin‐fixed and paraffin‐embedded tissues were retrieved from our archives and analyzed for the histopathological grade (whether high‐ or low‐grade) and the occurrence of heterologous differentiation. Then, tissues were processed routinely and two samples were chosen: one representative of the tumor core and one representative of the tumor periphery (up to 5 mm to the tumor margin). From these, tissues were cut into 0.5 μm slices, fixed on slides, and stained with hematoxylin‐eosin. Sections of 3 μm were used for immunohistochemical reactions according to standard techniques. The polyclonal rabbit antibody to CD117 identified all activated mast cells (Dako, Santa Clara, CA; c‐Kit clone, dilution 1:1000), the monoclonal mouse antibody to CD31 was used as a marker for endothelial cells (Dako; JC70A clone, dilution 1:800), and the mouse monoclonal antibody against Ki‐67 was used as a marker for LI (Dako; MIB‐1 clone, dilution 1:700). Positive controls for immunohistochemical reactions were done.

### 2.3 Image acquisition and digital image analysis

All immunostained tissue sections were scanned and analyzed by using an Aperio ScanScope X Slide Scanner (Aperio Technologies, Vista, CA) with a 20‐fold magnification (0.5 mμ resolution). According to manufacturer's manual, the computer‐based algorithm performed quantification automatically and results were given as a percentage of labeled cells (%) for mast cells and LI count. Digital imaging analysis was performed in all histological sections and presented as an average of the whole tumor specimen, considering the tumor core and the periphery separately. CD117 stained specimens were analyzed for MCD, while CD31 was applied to quantify MVD. It is worth noting that only vessels with a defined lumen were considered, the remaining ones were manually excluded from the measurement. MVD results were expressed as the number of vessels per square millimicron (vessels/mμ^2^).

### 2.4 Statistical analysis

Binary, ordinary, and categorical variables were compared by using chi‐square analyses (NF1 status, tumor location, tumor size, clinical staging, type of resection, and death), while continuous variables were compared using Mann‐Whitney non‐parametric test (age, MCD, MVD, and LI). For correlation analyses, the Pearson coefficient was applied. MCD, MVD, and Ki‐67 LI were categorized into terciles according to data distribution. Survival analyses were calculated by the Kaplan‐Meyer method and compared by the Mantel‐Haenszel log rank test. Cox proportional risk regression models evaluated each prognostic factor and potential combinations. To assess the robustness of our results, different models were developed, including MCD and MVD at the tumor core, at the periphery and both on the same model. We also tested models excluding the intermediate MVD tercile. The level of significance was set at *P* < 0.05. We used the R software (version 2.15.2; available at www.r-project.org) for all statistical analyses.

## 3 RESULTS

Out of the 92 patients admitted, 69 patients underwent surgery and 23 were transferred for palliative care. Other 40 patients were excluded due to neoadjuvant RT (n = 19) and incomplete histopathological records (n = 21). The excluded patients had a mean age of 42.41 ± 22.63 years (range 2‐84 years), 55.5% had NF1, and 54.5% were affected by large tumors (>10 cm).

Twenty‐nine patients (34 tumors) fulfilled our criteria and comprised our study population. Seventeen patients were male (58.6%), and patients affected by NF1 were generally younger than those having sporadic tumors (40.83 ± 10.53 and 50.24 ± 16.42 years, respectively). Twelve patients (41%) had 17 NF1‐associated MPNSTs (two patients had two tumors; one patient had four tumors). The remaining 17 tumors were sporadic. All tumors were high‐grade MPNSTs and none presented heterologous differentiation. We had no patient with RT‐induced MPNSTs. Demographic and clinical characteristics of the participants are presented in Table 1. Groups were fairly similar according to clinical characteristics, except death, which was marginally significant (NF1‐associated MPNSTs [100%], sporadic MPNST [71%]; *P* = 0.058).

**Table 1:** Main clinical characteristics in malignant peripheral nerve sheath tumors

Characteristic	NF1 (n = 12; 17 tumors)		Sporadic (n = 17; 17 tumors)		*P*‐value
Mean age (y, SD, range)	40.83 ± 10.53 (24‐55)		50.24 ± 16.42 (22‐83)		0.121
	n	%	n	%	
Tumor site					0.197
Head and neck	1	5.9	2	11.8	
Trunk	7	41.2	3	17.6	
Extremity	9	52.9	10	58.8	
Tumor size					0.492
<5 cm	3	17.6	3	17.6	
5‐10 cm	2	11.8	5	29.5	
>10 cm	12	70.6	9	52.9	
Stage					0.307
IIA	—	—	3	17.6	
IIB	2	16.7	3	17.6	
III	9	75	11	64.8	
IV	1	8.3	—	—	
Grade of resection					0.694
Total	9	75	11	64.8	
Subtotal	3	25	6	34.2	
Death					0.058
Yes	12	100	12	70.5	
No	—	—	5	29.5	

NF1, neurofibromatosis type 1.

### 3.1 MCD and distribution within MPNSTs

**Figure 1:**
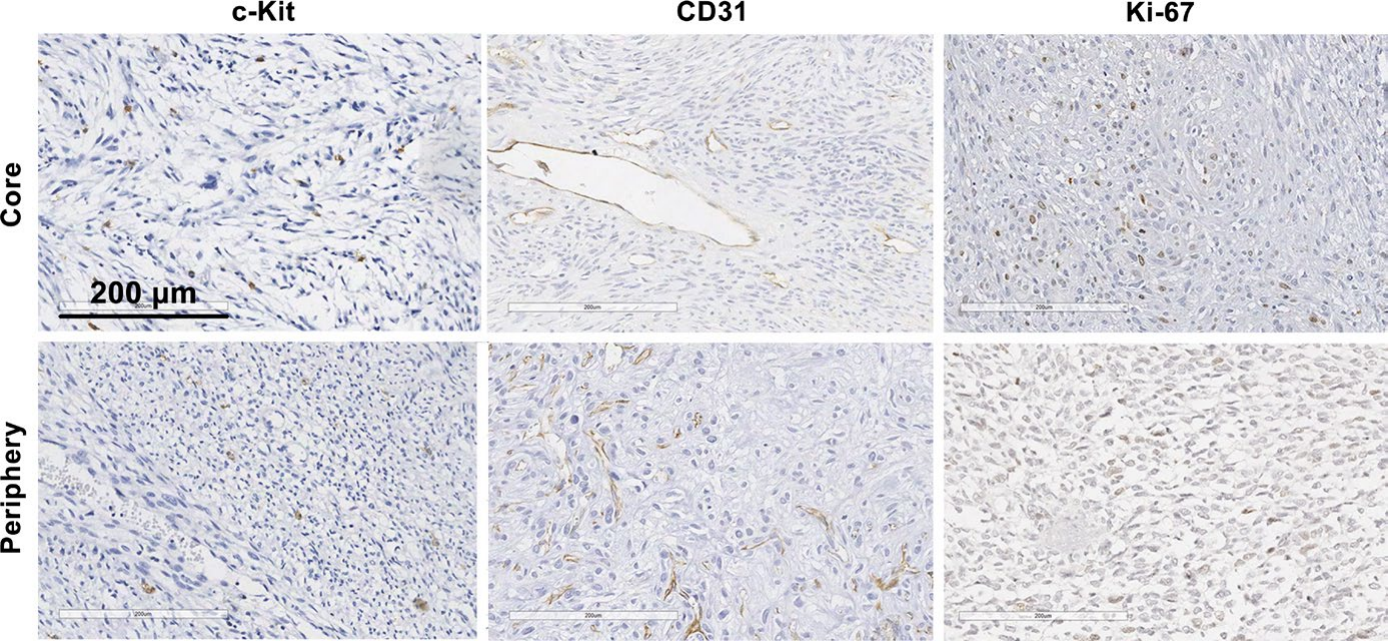
Overall, malignant peripheral nerve sheath tumors showed similar levels of c‐Kit, CD31, and Ki‐67 staining in the tumor core and periphery. Scale bar is equal to 200 μm

The prevalence of mast cells was evenly distributed throughout tumor core and periphery. There was no clustering of mast cells around specific structures within the tumor. The mean MCD for the whole group was 2.19 ± 2.03% (range, 0.19‐8.96) in the tumor core and 2.67 ± 3.62% (range, 0.27‐15.27) in the periphery of MPNSTs. Density and distribution of mast cells were similar between NF1‐associated and sporadic MPNSTs (Table 2; Figure 1).

**Table 2:** Immunohistochemical findings in malignant peripheral nerve sheath tumors

Marker	Core Mean ± SD (range)	Periphery Mean ± SD (range)	*w*	*P*‐value
Total				
MCD (%)	2.19 ± 2.03 (0.19‐8.96)	2.67 ± 3.62 (0.27‐15.27)	5.75	0.97
MVD (vessels/mµ^2^)	175.6 ± 62.97 (75.05‐346.7)	197.6 ± 58.39 (104.8‐352.3)	365.5	0.07
Ki‐67 (%)	7.65 ± 11.96 (0.08‐48.94)	5.84 ± 8.33 (0.04‐44.09)	540.5	0.7
NF1				
MCD (%)	2.07 ± 1.7 (0.2‐5.13)	2.11 ± 1.46 (0.27‐5.77)	132.5	0.68
MVD (vessels/mµ^2^)	198.1 ± 67.68 (106.6‐346.7)	215.1 ± 69.45 (104.8‐352.3)	108.5	0.47
Ki‐67 (%)	7.87 ± 12.35 (0.27‐48.94)	6.78 ± 11.14 (0.47‐44.09)	140	0.45
Sporadic				
MCD (%)	2.31 ± 2.37 (0.19‐8.96)	3.23 ± 4.92 (0.29‐15.27)	154	0.76
MVD (vessels/mµ^2^)	151.6 ± 48.86 (75.05‐284.3)	180 ± 39.72 (135.3‐309.3)	67	**0.037**
Ki‐67 (%)	7.43 ± 11.95 (0.08‐48.06)	5.03 ± 4.93 (0.04‐15.45)	133.5	0.93

	NF1 (mean)	Sporadic (mean)	NF1 (mean)	Sporadic (mean)		
MCD (%)	2.07 ± 1.7	2.31 ± 2.37	—	—	149	0.89
MVD (vessels/mµ^2^)	198.1 ± 67.68	151.6 ± 48.86	—	—	190	0.053
Ki‐67 (%)	7.87 ± 12.35	7.43 ± 11.95	—	—	134	0.84
MCD (%)	—	—	2.11 ± 1.46	3.23 ± 4.92	180	0.22
MVD (vessels/mµ^2^)	—	—	215.1 ± 69.45	180 ± 39.72	149	0.14
Ki‐67 (%)	—	—	6.78 ± 11.14	5.03 ± 4.93	116	0.68

MCD, mast cell density; MVD, microvascular density; NF1, neurofibromatosis type 1.

Bold value emphasize statistical significance.

### 3.2 MVD and distribution within MPNSTs

Regarding MVD, the number of vessels was higher in the periphery, but did not reach statistical significance when evaluated the entire population (197.6 ± 58.39 and 175.6 ± 62.97 vessels/mμ^2^, respectively). Subgroup analyses demonstrated that the number of vessels was clearly higher in the periphery of sporadic tumors in comparison to the tumor core (mean of 180 vessels/mμ^2^ and mean of 152 vessels/mμ^2^, respectively) (*P* = 0.037). MVD was similarly distributed throughout periphery (*P* = 0.14) and core (*P* = 0.053) of NF1‐associated MPNSTs and sporadic tumors (Table 2; Figure 1).

### 3.3 Proliferative Index and distribution in MPNSTs

The mean overall Ki‐67 LI was 7.65 ± 11.96% in the tumor core and 5.87 ± 8.33% in the periphery. LI showed similar levels in the core and periphery of NF1‐associated MPNSTs and sporadic tumors (Table 2; Figure 1).

### 3.4 Correlation with clinical characteristics and survival

Even though we found higher MVD at the periphery of sporadic MPNSTs, there was no correlation between MVD, MCD, LI, tumor distribution, or NF1 status (Table 3). When analyzed by tumor size, tumors greater than 10 cm had higher MVD both in the tumor core and periphery, but not in a significant level.

**Table 3:** Correlations between immunohistochemical findings in malignant peripheral nerve sheath tumors

Correlations	Core		Periphery	
	*r*	*P*‐value	*r*	*P*‐value
Total				
MVD × Ki‐67	−0.07	0.68	−0.2	0.28
MVD × MCD	−0.07	0.7	−0.1	0.6
Ki‐67 × MCD	−0.25	0.16	0.0007	0.99
NF1				
MVD × Ki‐67	−0.19	0.49	−0.27	0.35
MVD × MCD	−0.31	0.23	−0.09	0.77
Ki‐67 × MCD	−0.25	0.35	−0.32	0.23
Sporadic				
MVD × Ki‐67	0.05	0.86	−0.17	0.54
MVD × MCD	0.18	0.5	−0.06	0.89
Ki‐67 × MCD	‐0.26	0.32	0.24	0.35

MCD, mast cell density; MVD, microvascular density; NF1, neurofibromatosis type 1.

Median OS was 28 months for combined NF1‐associated/sporadic MPNSTs. In the univariate analysis, patients with NF1 had a significant reduction in the OS from 57 to 14 months (hazard ratio [HR] 2.44 [95% CI 1.06‐5.6, *P* = 0.036]), when compared to sporadic tumors. Increasing tumor was also associated with a worse outcome in the way that tumors greater than 10 cm had an OS of 16 months, while tumors smaller than 10 cm had an OS of 60 months (HR 3.24 [95% CI 1.18‐8.88, *P* = 0.023]).

**Figure 2:**
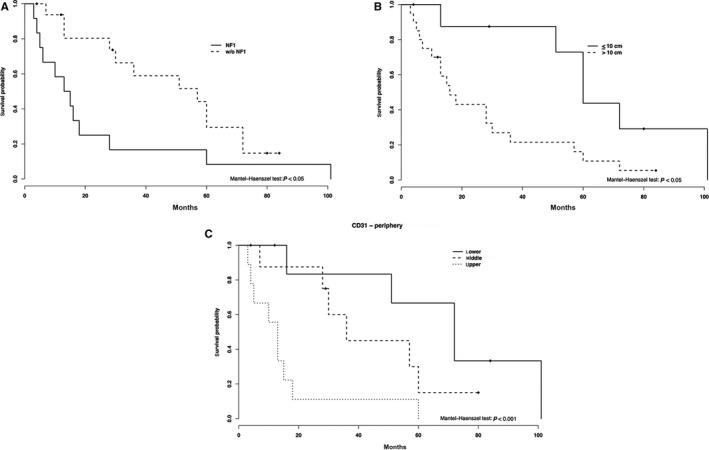
Survival curves by NF1 status (A) tumor size (B) and CD31 (C) levels in the tumor periphery (lower, middle, and upper terciles). NF1, neurofibromatosis type 1

The decreased OS for patients having tumors with MCD at the intermediate tercile at the periphery approached statistical significance (HR 2.54 [95% CI 0.95‐6.75, *P* = 0.062]). Higher MVD was associated with a worse OS (13 months), especially those patients having tumors with MVD at the higher tercile in tumor periphery (HR 8.22 [95% CI 2.19‐30.81, *P* = 0.002]) (Table 4). Conversely, Ki‐67 LI has shown no correlation to outcome. Survival analyses for NF1, tumor size, and MVD (tumor periphery) are shown in Figure 2.

**Table 4:** Univariate analysis of predictors of overall survival

Variable	Overall survival (median, mo)	HR (95% CI)	*P*‐value
NF1 status			
No (sporadic)	57	Ref	
Yes	14	2.44 (1.06‐5.60)	0.036
Tumor size			
<10 cm	60	Ref	
>10 cm	16	3.24 (1.18‐8.88)	0.023
MCD core (%)			
Lower tercile	15	Ref	
Interm. tercile	28	1.07 (0.40‐2.86)	0.893
Upper tercile	57	0.66 (0.22‐1.98)	0.458
MCD periphery (%)			
Lower tercile	30	Ref	
Interm. tercile	15	2.54 (0.95‐6.75)	0.062
Upper tercile	60	0.58 (0.19‐1.80)	0.350
MVD core (vessels/mµ^2^)			
Lower tercile	60	Ref	
Interm. tercile	36	1.77 (0.60‐5.20)	0.301
Upper tercile	10	2.20 (0.75‐6.42)	0.151
MVD periphery (vessels/mµ^2^)			
Lower tercile	72	Ref	
Interm. tercile	36	2.11 (0.57‐7.81)	0.265
Upper tercile	13	8.22 (2.19‐30.81)	0.002
Ki‐67 core (%)			
Lower tercile	18	Ref	
Interm. tercile	21.5	1.01 (0.33‐3.11)	0.983
Upper tercile	51	0.83 (0.31‐2.23)	0.710
Ki‐67 periphery (%)			
Lower tercile	29	Ref	
Interm. tercile	57	1.40 (0.45‐4.39)	0.562
Upper tercile	60	2.26 (0.78‐6.56)	0.133

HR, hazard ratio; Interm., intermediate; MCD, mast cell density; MVD, microvascular density; NF1, neurofibromatosis type 1; ref, reference.

Multivariate analysis confirmed that only MVD in tumor periphery (higher tercile) was associated with a decreased OS, increasing the risk of death by approximately seven times (HR 7.28 [95% CI 1.38‐38.46, *P* = 0.019]) (Table 5).

**Table 5:** Multivariate analysis of predictors of overall survival

Variable	HR (95% CI)	*P*‐value
NF1 status (yes)	0.86 (0.22‐3.42)	0.827
Tumor size (>10 cm)	2.27 (0.66‐7.82)	0.196
MVD periphery interm. tercile (vessels/mµ^2^)	2.32 (0.59‐9.17)	0.230
MVD periphery upper tercile (vessels/mµ^2^)	7.28 (1.38‐38.46)	0.019

HR, hazard ratio; Interm., intermediate; MVD, microvascular density; NF1, neurofibromatosis type 1.

## 4 DISCUSSION

Our study provides evidence about MPNST microenvironment as it shows a relatively similar distribution of MCD, Ki‐67 LI, and MVD throughout the tumor in whole tissue sections. Sporadic MPNSTs comprised the only exception because of the marked neovascularization in the periphery. Correlation studies with clinical variables confirmed that NF1 status, tumor size (>10 cm), and MVD in tumor periphery (higher tercile) lead to a significant reduction of the OS. In the multivariate analysis, only higher MVD in tumor periphery retained an independent prognostic significance in the studied population.

Since the 1990 s, mast cells were shown to play a role in the formation of neurofibromas, when distinct groups of researchers found a much higher distribution in NF1‐associated and sporadic tumors compared to adjacent areas of normal skin.^40^, ^41^ Contemporary studies have contributed to this issue by demonstrating the essential participation of mast cells in the pathogenesis of plexiform neurofibromas in both animal models^33^ and humans.^34^

In this context, neurofibromin‐deficient Schwann cells (*Nf1 −/−*) secrete chemotactic factors (Kit ligand) to stimulate the migration of haploinsufficient mast cells (*Nf1 +/−*), which in turn can locally secrete neural growth factor (NGF) and vascular endothelial growth factor (VEGF) in the tumor microenvironment.^33^ Ultimately, this indicates the potential implication of the interaction of Schwann cells and mast cells and inflammation in the pathogenesis of neurofibromas. Furthermore, Tucker et al^34^ demonstrated that the distribution of mast cells was much more intense and dispersed in diffuse neurofibromas in relation to encapsulated neurofibromas. Such distribution could account for a different tumorigenesis in both tumors.^34^

Using manual counting in 16 neurofibromas, Tucker et al^34^ showed that mast cells represented 4.67 ± 3.95% of 5062 nuclei in diffuse neurofibromas and 0.73 ± 0.97% of 4186 nuclei in encapsulated neurofibromas. It should be noted that these values were determined with toluidine blue labeling, which represents the subpopulation of non‐activated mast cells. The number of activated mast cells marked by c‐Kit staining was significantly higher, especially in diffuse neurofibromas. The authors did not describe the absolute values, however.^34^ Additionally, Friedrich et al^35^ studied 124 benign peripheral nerve tumors and eight MPNSTs revealing a consistently different MCD in diverse tumors. Mast cells were stained with the periodic acid Schiff and counted manually in four fields of vision of 0.03 mm^2^ each. MCD was significantly lower in MPNSTs, with a median distribution of 25 to 50 mast cells/mm^2^. It is worth noting that MCD in MPNST was lower than control tissues.^35^

Recently, Dodd et al^36^ confirmed in experimental models and human tissues that elevated levels of mast cells developed NF1‐associated MPNSTs at an accelerated rate. Quantification was done with toluidine blue staining (sum of six fields for a single slide) for mice models and c‐Kit immunohistochemistry for human tissues (<5, 5‐50, and >50 mast cells). Importantly, NF1‐associated tumors had significantly more mast cells than sporadic ones (more than 5 cells in about 50% and 30%, respectively).^36^ Although it is not possible to directly compare these studies because of different counting and immunolabeling methods, our results corroborate the low MCD in MPNSTs, as demonstrated by Friedrich et al,^35^ indicating that mast cells are potentially less relevant in the progression of malignant tumors. In addition, our work also shows that MCD lacks prognostic value.

Generally speaking, c‐kit expression is the most commonly used staining technique to study the role of mast cells in peripheral nerve sheath tumors,^34^, ^35^, ^36^ certainly because c‐kit driven mast cell activation promotes the release of tryptase, the most important pro‐angiogenic factor.^42^, ^43^ Canine models confirmed such assumption by demonstrating the correlation of c‐kit expression to the degranulated status on mast cells.^43^ For breast cancer, mast cell staining for c‐kit and tryptase are essentially the same due to an almost perfect correlation suggesting that c‐kit expression represents tryptase activation. Such observation has a potential therapeutic role in the way that it could characterize a novel anti‐angiogenic target for either c‐kit receptor or tryptase inhibitors in patients having elevated c‐kit expression.^42^

Similar to MCD, Ki‐67 LI shows a relatively similar distribution in tumor core and periphery regardless of the NF1 status. In addition, it has no correlation with the outcome. Our findings are not in line with those presented by Watanabe et al,^44^ in which tumors with LI greater than 25% have a worse prognosis. It is worth mentioning that only two patients in our study showed LI greater than 25%, nonetheless without impact in the OS. Our findings corroborate the studies by Brekke et al^45^ and Zou et al,^5^ who also did not observe LI as an important prognostic factor. Moreover, MCD has no correlation to LI or MVD suggesting that MPNSTs progression and neoangiogenesis may occur independently from mast cells distribution. We did not find any literature to compare our findings.

Finally, the literature is relatively scarce with few studies published so far on MVD in neurofibromas and MPNSTs. Tucker et al^34^ used a manual semi‐quantitative method to count vessels without immunohistochemical analysis in diffuse and encapsulated neurofibromas. Blood vessels were equally distributed throughout tumors regardless of tumor type.^34^ Friedrich et al^35^ analyzed MVD stained with CD34 in an automated way using six adjacent tumor areas with a total estimated area of 2.5 mm^2^. Malignant tumors exhibited significantly marked vascularization (30.8 vessels/mm^2^) in comparison to benign tumors (13.46 vessels/mm^2^).^35^ Again, different counting and labeling methods does not allow a direct comparison between our results and those previously obtained in the literature. On the other hand, we observed that MPNSTs with greater MVD in the periphery carry a worse prognosis, an issue previously demonstrated in astrocytomas,^46^ glioblastomas,^47^ prostate carcinomas,^48^ and colorectal carcinomas.^49^

### 4.1 Limitations

Our study has some key limitations. First, we have conducted a retrospective single‐center cohort analysis. In that way, data collection and characterization of patients are prone to inaccuracies and inconsistencies over the course of the study. Second, about two‐thirds of the patients were excluded from our evaluation because of advanced disease, neoadjuvant RT, and unavailable histopathological data. This could raise a question of whether our results are representative of the entire population. Considering similar epidemiological characteristics, age and NF1 status between the studied and the excluded population, we believe our findings are representative. It should be noted, however, that this is a rare disease, and we present herein the largest study on the investigation of neovascularization and mast cell distribution for MPNSTs.

## 5 CONCLUSIONS

Diverse mast cell and microvascular distribution suggest that angiogenesis in MPNST occurs independently and mast cells are potentially not involved in tumor progression. Besides, MCD lacks prognostic value. NF1 status and increasing tumor size remain the most important predictors of OS in MPNSTs in our population. Higher MVD was correlated to tumor size and has prognostic significance with possible therapeutic implications.
